# Material Characterization of Magnetorheological Elastomers with Corroded Carbonyl Iron Particles: Morphological Images and Field-dependent Viscoelastic Properties

**DOI:** 10.3390/ijms20133311

**Published:** 2019-07-05

**Authors:** Siti Aishah Binti Abdul Aziz, Saiful Amri Mazlan, Nur Azmah Nordin, Nor Azlin Nazira Abd Rahman, U Ubaidillah, Seung-Bok Choi, Norzilawati Mohamad

**Affiliations:** 1 Engineering Materials and Structures (eMast) iKohza, MJIIT, Universiti Teknologi Malaysia, Jalan Sultan Yahya Petra, Kuala Lumpur 54100, Malaysia; 2 Advanced Vehicle System (AVS) Research Group, Malaysia—Japan International Institute of Technology (MJIIT), Universiti Teknologi Malaysia (UTM), Kuala Lumpur 54100, Malaysia; 3 Department of Mechanical Engineering, Faculty of Engineering, Universitas Sebelas Maret, Surakarta 57126, Indonesia; 4 National Center for Sustainable Transportation Technology (NCSTT), Bandung 40132, Indonesia; 5 Department of Mechanical Engineering, Inha University, 253, Yonghyun-dong, Namgu, Incheon 22212, Korea

**Keywords:** magnetorheological elastomer, hydrochloric acid, particle corrosion, morphology observation, hydrangea-like structure, rheology, rubber

## Abstract

High temperatures and humidity could alter the field-dependent rheological properties of MR materials. These environmental phenomena may accelerate the deterioration processes that will affect the long-term rheological reliability of MR materials such as MR elastomer (MRE). This study therefore attempts to investigate the field-dependent rheological characteristics of MRE with corroded carbonyl iron particles (CIPs). The corroded CIPs were treated with hydrochloric acid (HCl) as a way of providing realistic environments in gauging the CIPs reaction towards the ambient conditions. The corroded CIPs along with silicone rubber as a matrix material were used in the fabrication of the MRE samples. To observe the effect of HCl treatment on the CIPs, the morphological observations of MREs with non-corroded and corroded CIPs were investigated via field emission scanning electron microscopy (FESEM), energy-dispersive x-ray spectroscopy (EDX) and x-ray diffractometer (XRD). In addition, the magnetic properties were examined through the vibrating sample magnetometer (VSM), while the field-dependent rheological characteristics such as the storage modulus of MRE with the corroded CIPs were also tested and compared with the non-corroded CIPs. The results showed that the corroded CIPs possessed hydrangea-like structures. In the meantime, it was identified that a sudden reduction of up to 114% of the field-dependent MR effect of MRE with the corroded CIPs was observed as a result of the weakened interfacial bonding between the CIPs and the silicon in the outer layers of the CIPs structure.

## 1. Introduction

Magnetorheological elastomer (MRE) possesses excellent rheological properties in which their stiffness can be controlled continuously and reversibly in the presence of an external magnetic field. MREs consist of micron size of magnetic particles, particularly Fe or carbonyl iron particles (CIPs) embedded in a non-magnetic matrix. Generally, natural rubber (NR) [1,2], silicon rubber (SR) [3,4], epoxidized natural rubber (ENR) [5] and thermoplastic [6] are utilized as matrix materials in the fabrication of an MRE. During the last two decades, MREs had been receiving considerable attention from both the academy and various fields industry owing to their enormous potential in engineering applications, particularly in the research and development of MRE devices such as bushing [7], vibration isolators [8], base isolators [9] and sensing devices [10,11,12].

Conventional rubber components such as vibration isolator and damper are frequently used for a long period of time and are subjected to various temperatures or/and under severe environmental conditions such as high oxygen, water, and oil contamination exposure. As a result of being exposed to these conditions, the rubber in engineering components may suffer from environmental degradation. The exposure can profoundly affect its level of performance [13] by changing the quality and characteristics of the material components, hence shortening its useful operation time [14,15,16,17,18]. Zhu et al. [16] fabricated two types of rubber, namely hydrogenated nitrile-butadiene rubber (HNBR) and nitrile rubber (NB), in order to investigate the effects of CO_2_ to the corrosion phenomenon under actual downhole condition. They found that the hardness of the rubbers decreased by up to 15% under a compression state for 168 h. Morphological changes were also observed with the appearance of shallow and small-sized holes on the surface of the HBNR. Sarlin et al. [17] had investigated the environmental resistance of stainless steel/rubber/glass fiber reinforced epoxy (GFRP) hybrid structures under the exposure of hot, moist and hot/moist environments. In this particular test, the researchers used different grades of EPDM-based rubber with the following components; polyethylene wax (rubber A), silica (rubber B) and carbon black (rubber C). After having undergone the process of hygrothermal exposure (85 °C, 85%RH), some minor degradations were observed in rubbers B and C at 370 °C, while rubber A showed symptoms of degradation at 500 °C. At the same time, the hardness level of rubbers B and C was increased by 5%–7% as compared to rubber A. In another study, Ozawa et al. [18] discovered a weakened interfacial adhesion that was caused by the increased of oxygen composition in the rubber-brass interface. Thus, for this reason, study on the degradation of material properties, as a result of hygrothermal aging or under practical conditions, is essential in the designing stage of material structures.

Nonetheless, magnetic particles such as Fe, Ni, Co and NdFeB are widely used in various fields such as electronics, acoustics, automation and biomedical applications. Song et al. [19] found HNO_3_ to be the strongest corrosion electrolyte that obviously accelerated the corrosion of NdFeB in their study about corrosion behavior in various electrolyte solutions such as NaOH, NaCl, HNO_3_, and H_2_C_2_O_4_. Pores and crevices were observed in the morphology of NdFeB after being immersed for 0.5 h in HNO_3_, hence implying a degradation of magnetic properties from the hydrogen released in the magnets’ surface. On the other hand, Dunn et al. [20] proposed in-situ alternating current (AC) and direct current (DC) electrochemical techniques in determining the corrosion rate and corrosion potential of high-purity iron under alternative wet and dry states.

It is a well-known that magnetic particles are the main component in the MR materials such as MR fluid, MR grease, MR foam and MRE. The study on the effect of corroded carbonyl iron particles (CIPs) in MR fluid has been reported by a few researchers. Han et al. [21] studied the effect of corroded CIPs on the performance of MR fluids, particularly in the field-dependent properties such as the yield stress. They found that the shear stress of MR fluid with the corroded CIPs decreased up to 11% and with a slower response time than those of the non-corroded MR fluid. In studying the influence of 0.05 HCl on the corrosion of CIPs in MR fluid, Plachy et al. [22] discovered the formation of the Fe_2_O_3_ oxide layers on the surfaces of the particles, while the magnetic saturation of the oxidized CIPs decreased up to 35% more than non-oxidized CIPs. Recently, Cvek at al. [23] fabricated two types of CI particles grafted with poly(trimethylsilyloxyethyl methacrylate) (PHEMATMS) of two different molecular weights. The results revealed that the PHEMATMS did enhanced the stability of CI particles and prevented the degradation in an acidic condition.

As evident from the abovementioned literature, attention and majority of the published works was mainly focused to the influence of corroded CIPs solely in rheological properties of MR fluid. However, the effect and the influence of the corroded iron particles on the physicochemical and rheological, properties particularly the storage modulus and damping of MRE has not been reported so far. Thus, further investigation and the mechanism of the corroded CIPs effect toward the properties of MRE needs to be undertaken through both the morphological and rheological approaches. For this purpose, in this work an attempt is made to characterize and emphasize comprehensive physicochemical and rheological properties of MRE samples with non-corroded and corroded CIPs through empirical methods. Therefore, it is asserted that the main technical contribution of this work is to experimentally investigate the principal characteristics of MREs such as the field-dependent storage modulus by fabricating MRE samples containing the corroded CIPs. This research idea comes from the possibility that new iron particles containing in MRE may be corroded after long-time use of MRE under practical conditions. This investigation is closely related to the behavior of MRE applied under various environmental conditions and hence is a very significant area to be explored. In this study, hydrochloric acid (HCl) is used to produce corroded CIPs for the fabrication of silicone rubber based MRE samples. The correlation between microstructure evolution, magnetic behavior and rheological properties such as storage modulus, damping properties and MR effect of the corroded CIPs in MRE is systematically investigated.

## 2. Results and Discussions

### 2.1. Morphological Results and Discussions

#### 2.1.1. Morphological Properties

**Figure 1:**
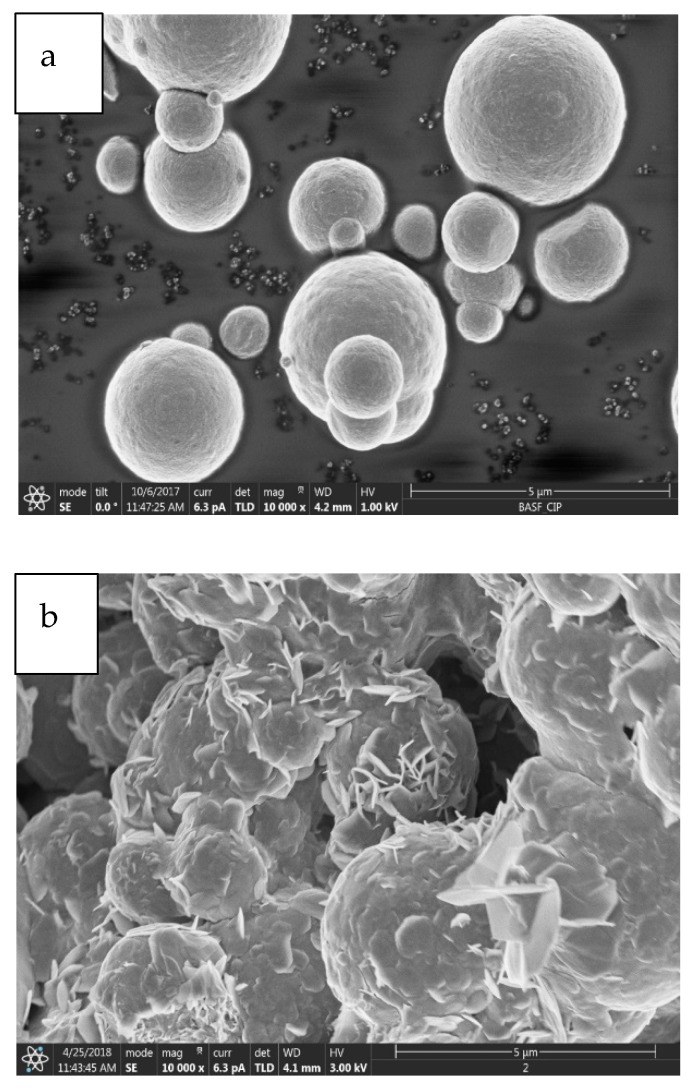

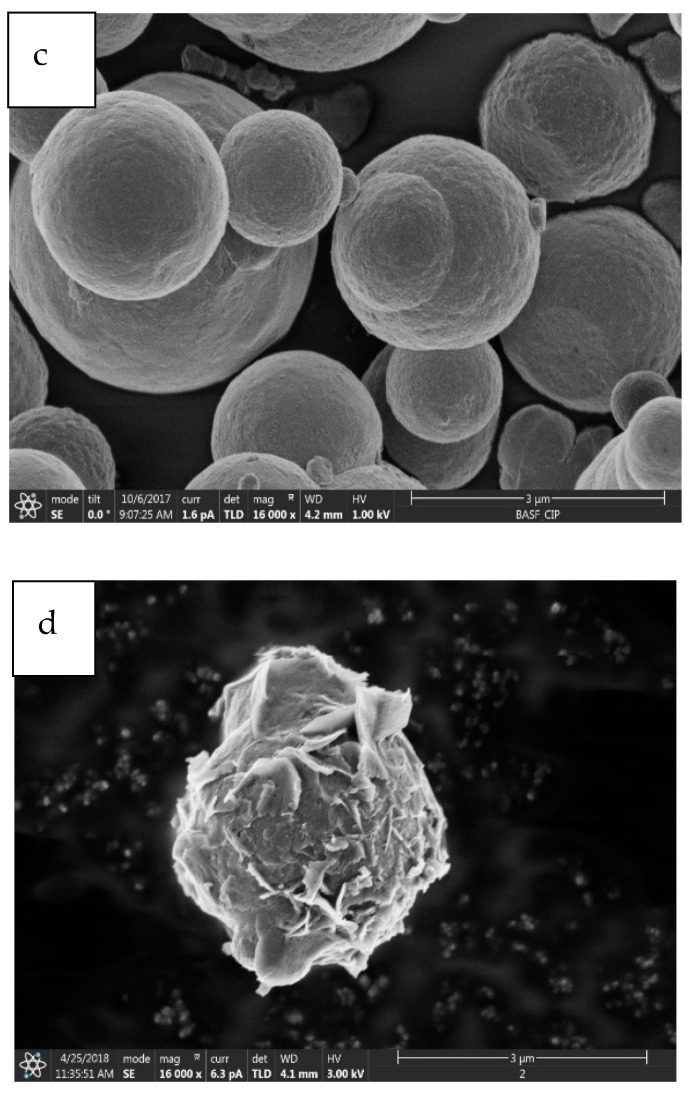
The CIPs samples of (**a**) non-corroded and (**b**) corroded CIPs at magnification of 10,000×, and (**c**) non-corroded and (**d**) corroded CIPs at magnification of 16,000×.

Figure 1 shows FESEM images of surface morphologies of spherical and corroded CIPs at 10k× and 16k× magnification, where the microstructures of non-corroded and corroded CIPs were observed.

The non-corroded CIPs initially demonstrated spherical and micrometer-sized features as shown in Figure 1a,c. Meanwhile, the microstructures of corroded CIPs changed to a hierarchical hydrangea-like structure like those in Figure 1b,d. The chemical reaction that occurred between the CIPs and the surrounding acidic condition had caused the formation of a corroded layer on the CIP’s surface structure. These outer layers might have contributed to the increment of particles size as well as its hard and brittle structures [24]. In Figure 1b, apart from the structure of the CIPs surface, the agglomerated portion of the CIPs was observed since the small CIPs displayed strong tendencies for attaching to one another in the cluster formation. In addition, the HCl solution might also cause the breakdown, cracking and flacking on the surface of CIPs particles. The rough surface of the corroded CIPs at a high magnification is shown in Figure 1d, suggests that a higher HCl concentration had dissolved into the surface of CIPs.

**Figure 2:**
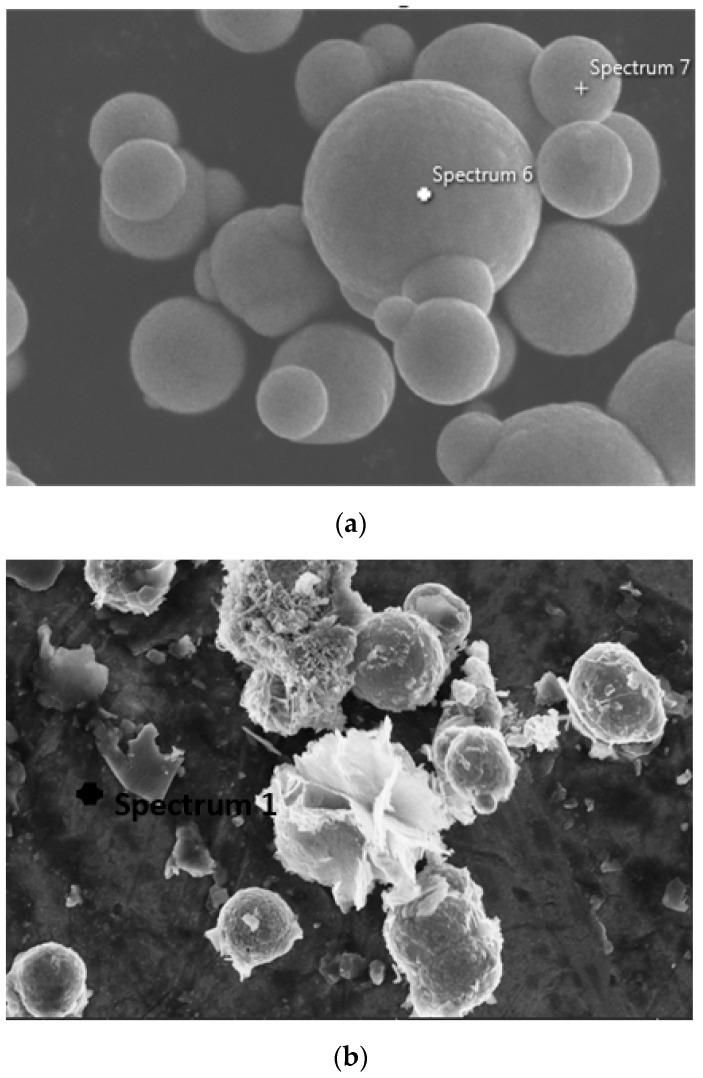
EDX point analysis at 10,000× magnification for (**a**) non-corroded and (**b**) corroded CIPs.

In order to study the changes that occurred in the surface characteristics of corroded CIPs, the FESEM-EDX analysis was then performed to substantiate the formation of the corroded layer on the surface of CIPs. Figure 2 shows point analysis before and after the exposure CIPs in the corrosive medium.

EDX analyses for the CIPs before and after the corrosion are embedded and summarized in Table 1. Table 1 exhibited that the CIPs are dominated by Fe and O. The result showed that after the corrosion, the O peak has increased, which implied that the O content in the corrosion product for this region was higher as compared to the non-corroded CIPs. Meanwhile, the Fe content decreased due to the oxidized layer that developed on the surface of the corroded CIPs.

The O content in the corroded CIPs increased by up to 22.94% more than in the non-corroded CIPs. This result occurred due to the exposure of CIPs to acid containing H^+^ from the diluted HCl as medium corrosion and its reaction with oxygen from the surrounding, which led to the reduction and oxidation (REDOX) reaction to form the iron rust.

#### 2.1.2. Phase Characterization

**Figure 3:**
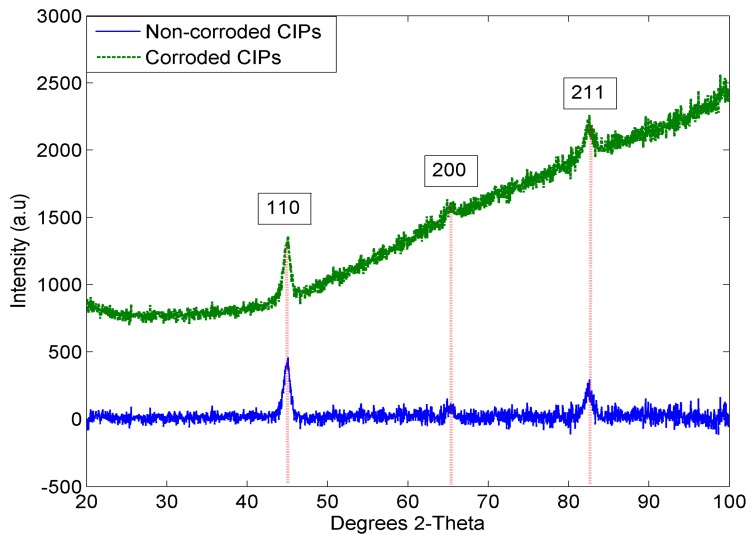
XRD patterns for corroded and non-corroded CIPs.

The XRD patterns of the corroded and non-corroded CIPs are shown in Figure 3. The patterns clearly showed one sharp and strong diffraction peak at 2θ = 44.97° for both MRE samples, which was assigned to (110) plane as a reflection of the Fe crystal plane. Another diffraction peak of Fe was also observed at 2θ = 82.45°, which could be assigned to (211) plane. Additionally, another small diffraction peak at around 65.22° corresponded to (200) plane of Fe (JCPDS card No. 04-0850) was seen, which agreed well with the values reported by other researchers [25,26,27,28].

The MRE with corroded CIPs sample has shown such increment pattern attributed to the crystalline phase changes and the changes in the lattice parameters. Due to the corrosion effect, the phase started to change from crystalline to amorphous. This result was consistent with the morphological finding in Figure 1b,d. The agglomeration occurred in the corroded CIPs has changed the atomic order of CIPs as compared to the pure CIPs. Thus, it was observed that the peak of intensity values increased due to crystal defects related to the surface roughness of the CIPs. In this result, no peak due to the impurities was observed.

#### 2.1.3. Vibrating Sample Magnetometer

**Figure 4:**
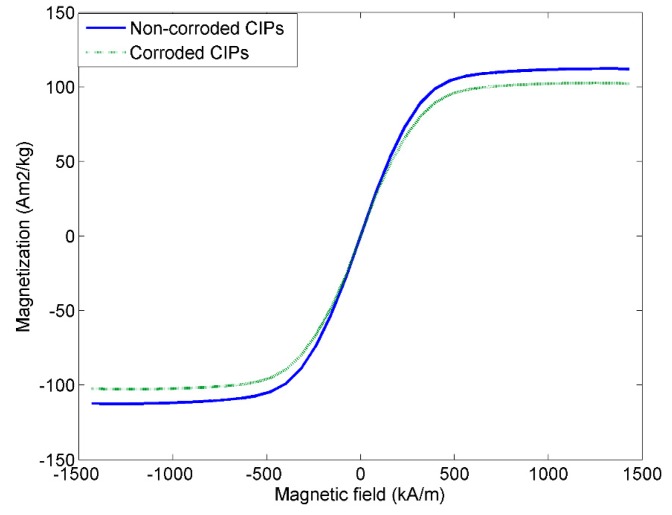
Magnetization curves for MRE samples of non-corroded and corroded CIPs.

Magnetic properties of MRE samples were evaluated using VSM. The hysteresis loops for the non-corroded and corroded CIPs, measured up to 1500 kA/m are shown in Figure 4. It is noted that both non-corroded and corroded CIPs displayed narrow magnetic hysteresis loops, which were related to the soft magnetic characteristics of the MRE samples.

The non-corroded CIPs has shown a higher magnetic saturation at 112.3 Am^2^/kg as compared to corroded CIPs at 102.2 Am^2^/kg. The saturation value was slightly difference might be due to the oxidation layer that formed on the surface of the CIPs. Meanwhile, other magnetic properties such as coercivity HC, and retentivity magnetization MR measured at room temperature are summarized in Table 2.

The remnant magnetization, M*r* of non-corroded and corroded CIPs were 1.30 and 1.28 Am^2^/kg, respectively. While the coercive force, H*c* of non-corroded and corroded CIPs were 0.70 and 0.68 kA/m, respectively. Nonetheless, the M*r* and H*c* values for corroded CIPs were slightly lower than non-corroded CIPs due to the loss of magnetic properties by the formation of oxide layers on the CIPs surface [29]. In an acidic condition, the chloride and iron reacted easily and chemically make a new interaction in CIPs. Meanwhile, it is well known that during the corrosion process, or in the presence of water or moisture in the air, the iron is reacted easily with the oxygen, which can be expressed in Equations (1)–(5).

Oxidation: Fe → Fe^2+^ + 2e^−^ (1)

Reduction: O_2_ + 4H^+^ + 4e^−^ → 2H_2_O (2)

Corrosion: 2Fe + O_2_ + 4H^+^ → 2Fe^2+^ + 2H_2_O (3)

2Fe^2+^ + 4OH^-^ + 2H^+^ → 2Fe(OH)_2_ + H_2_ (4)

Further oxidized: 4Fe(OH)_2_ + O_2_ + H_2_O → 2Fe_2_O_3_·xH_2_O (5)

The interactions between CIPs and oxygen during the corrosion process will end up with the reduction of the magnetic properties.

### 2.2. Rheological Results and Discussions

#### 2.2.1. Frequency Sweep Test

**Figure 5:**
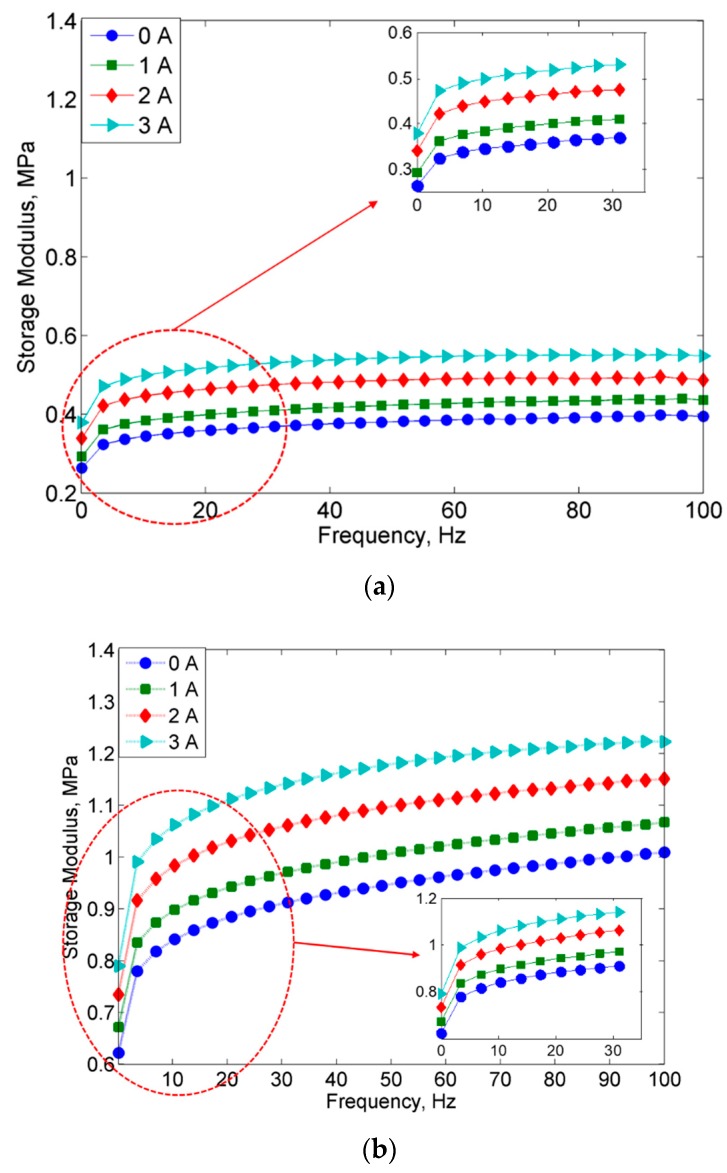
Storage modulus vs. frequency of (**a**) MRE with non-corroded CIPs and (**b**) MRE with corroded CIPs at different applied currents.

Figure 5 presents the results of storage modulus (G’) of MRE samples that were obtained from the frequency sweep tests at different magnetic field strengths by varying applied currents of 0, 1, 2 and 3A.

It could be observed that the storage modulus for both MRE samples had increased as the frequency and magnetic field increased. More specifically, in Figure 5a, the initial storage modulus for MRE sample containing non-corroded CIPs has increased with the increase of magnetic field, which was from 0.26, 0.29. 0.34 to 0.39 MPa at 0, 1, 2 and 3A, respectively. At the same time, similar trend with the initial storage modulus, the maximum storage modulus of MRE with non-corroded CIPs was valued 0.38, 0.43, 0.49 and 0.55 MPa at 0, 1, 2 and 3A. Nonetheless, the storage modulus for MRE samples with corroded CIPs, as shown in Figure 5b, at off-state condition has increased from 0.62 MPa to the maximum storage modulus of 1.01 MPa as the frequency increases from 0.1 at 100 Hz. The summary of the initial and maximum storage modulus is depicted in Table 3.

As shown in Table 3, in general, at all applied magnetic fields, the MRE with non-corroded CIPs exhibited much lower in storage modulus, in fact, almost half of those values from MRE with corroded CIPs. For example, at a constant applied current of 3 A, the initial storage modulus of MRE with corroded CIPs was at 0.79 MPa, while non-corroded CIPs was at 0.39 MPa. Thus, the results showed that about 100% increase in the initial storage modulus by implying a higher stiffness property of the MRE with corroded CIPs. The increment trend of storage modulus in MRE with corroded CIPs might be due to the different surface contacts between the silicon with corroded CIPs. As for the MRE with corroded CIPs, it is believed that during the early stage of curing condition, the liquid silicone would penetrate to the rough surface of CIPs in which it was cured in that condition. Apparently, the interaction of CIPs and HCl acid during the immersion causes CIPs agglomeration created a better interaction of the filler-filler (CIPs), which resulted in a higher storage modulus with the existence of a magnetic field. This kind of phenomenon is similar to the one reported by Mostafa et al. [30] in which the CIPs did exhibit good wetting of particles due to the wettability effect of HCl and thus exhibited better CIPs interaction. In other words, this phenomenon was obtained due to stress relief by the particle rearrangement in the matrix-particle network of MRE with corroded CIPs, and hence affected the storage modulus of MRE [31]. Meanwhile, for the MRE with non-corroded CIPs, at high frequency, the change in the slope of the curves at higher magnetic fields was due to the magnetic forces between CIPs were lower due to increment of interparticle distance among CIPs. In the meantime, the increment of the interparticle among CIPs could also lead to an increment in molecular mobility after softening of MRE with an increase in frequency. 

**Figure 6:**
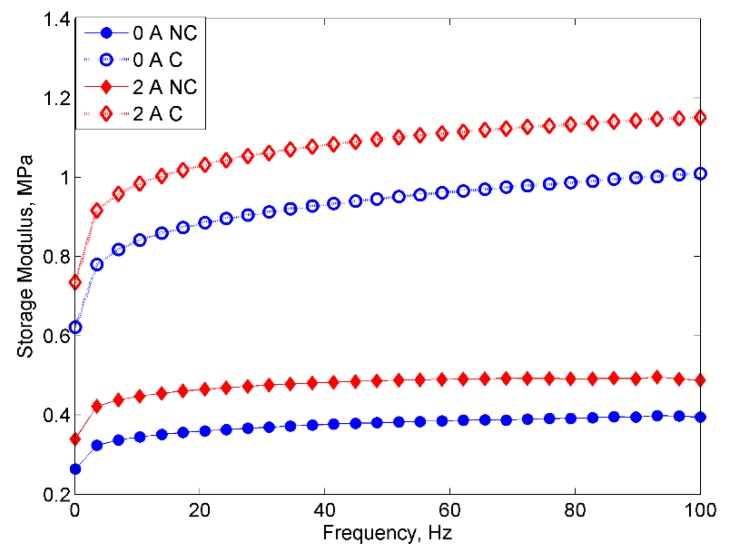
Storage modulus vs. frequency for MRE with non-corroded (NC) and corroded (C) CIPs at off- and on-state conditions.

Figure 6 shows the comparison of a storage modulus between two MRE samples at off- and on-state conditions.

It could be seen that the MRE sample with corroded CIPs exhibited a higher storage modulus at both off and on-state conditions as indicated by 138% and 114% increase in the storage modulus, respectively. It is noted here that the oxidation layer appeared on the corroded CIPs was mostly brittle and might lead to the decrement of its material properties, such as a storage modulus. Thus, it is believed that the chemical interaction of the corroded CIPs with regards to its agglomeration had resulted in strong bonds between the CIPs as a consequence of an accelerated electrochemical corrosion. In this case, the oxidation-reduction that occurred in the CIPs during the corrosion process enhanced the strength or possibly the stiffness level of the MREs [32], which was reflected by the increment of the storage modulus of MRE with corroded CIPs at off- and on-state conditions. The bonding charge of the CIP molecules was also strengthened by the influence of the magnetic field as well as from the effects of HCl, which had probably acted as a wetting agent during the corrosion process.

**Figure 7:**
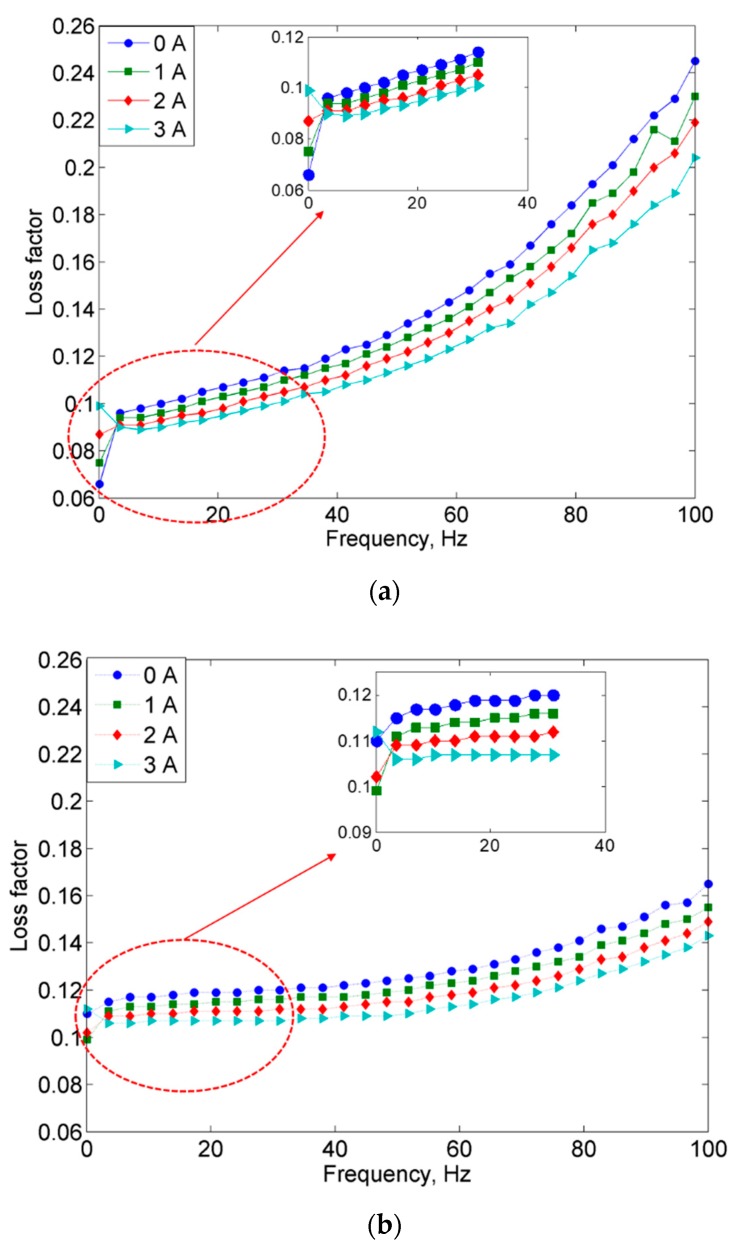
Loss factor vs. frequency for (**a**) MRE with non-corroded CIPs and (**b**) MRE with corroded CIPs.

Another important material property of the MREs is the field-dependent loss modulus or loss factor. The measured loss factor according to its respective applied frequencies is shown in Figure 7.

The loss factor decreased with an increasing level of the magnetic field but had been shown otherwise with increasing frequency. Nevertheless, it was observed that the MRE with non-corroded and corroded CIPs had experienced an increase of loss factor to above 10 Hz. Loss factor had been shown to strongly depended on the movement, deformation and alignment of the magnetic particles [3,16]. At low frequencies, the molecular chain underwent random rupture and became smaller, hence resulting in longer relaxation times, which might have contributed to the reduction in MRE’s loss factor. Meanwhile, at high frequencies, the higher friction could be caused by the restriction of particles’ movements. The lower loss factor of MRE with corroded CIPs as compared to MRE with non-corroded CIPs is believed to be due to the stiffer behavior that occurred in the MRE. Under this condition, the magnetic forces between the CIPs became stronger owing to the reducing of the interparticle distance among CIPs, which at the same time resulted in lowering the loss factor properties.

**Figure 8:**
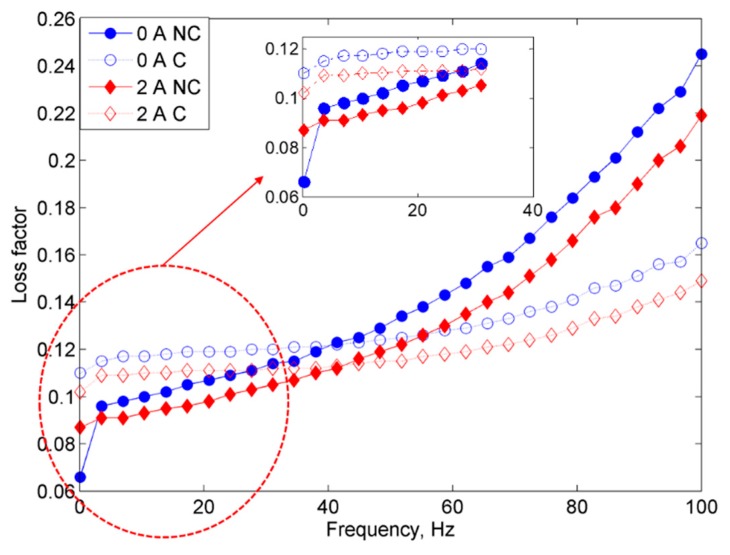
Comparison of loss factor vs. frequency for MRE samples of non-corroded (NC) and corroded (C) CIPs at off- and on-state conditions.

As shown in Figure 8, initially, MRE with corroded CIPs exhibited a lower loss factor with increasing frequency than those with non-corroded CIPs at constant currents of 0 and 2A. This phenomenon might be due to the higher surface area of CIPs that contributed by the oxidized layer on the CIPs, which in turn led to reduction of the internal friction due to a matrix-filler network breakdown. Thus, this kind of phenomenon resulted in lowering the loss factor.

#### 2.2.2. Strain Sweep Test

**Figure 9:**
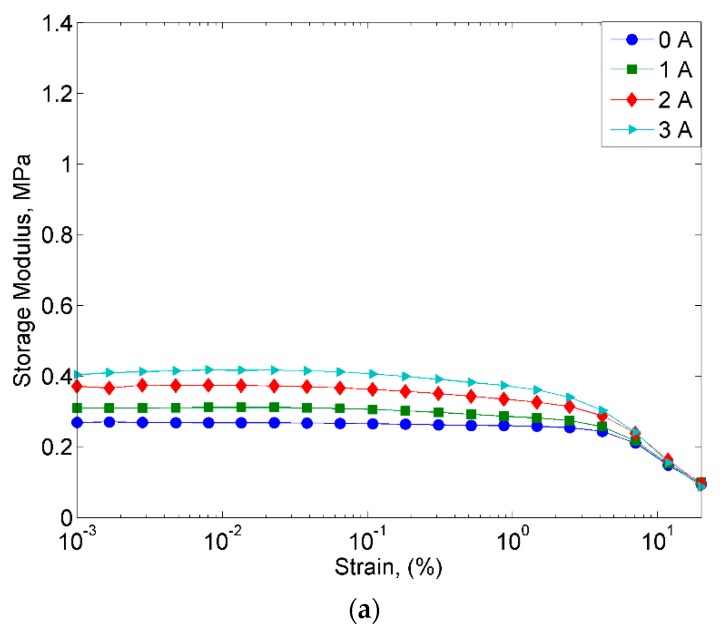

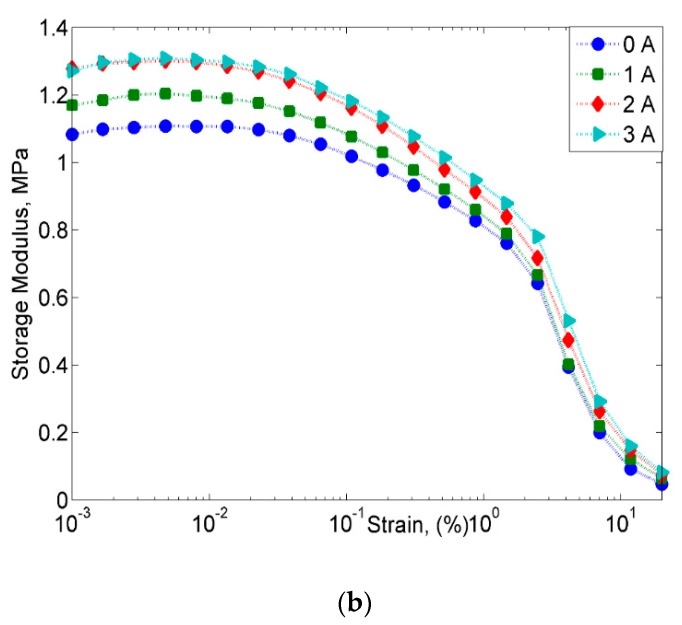
Storage modulus vs. strain for (**a**) MRE with non-corroded CIPs and (**b**) MRE with corroded CIPs.

The variation of the storage modulus towards sweep strain amplitude are shown in Figure 9. At all applied magnetic fields, both MRE samples exhibited an increasing trend at the initial storage modulus.

The initial storage modulus of both MRE samples are shown in Table 4. The initial storage modulus of MRE with non-corroded CIPs of 0, 1, 2, and 3A were observed at 0.26, 0.31, 0.37 and 0.40 MPa, while the equivalents for corroded CIPs were at 1.08, 1.17, 1.28 and 1.27 MPa. At an off-state condition, the initial storage modulus of MRE with corroded CIPs was three times higher (307%) than non-corroded CIPs.

The increment of the initial storage modulus of MRE with corroded CIPs has revealed that the sample was stiffer as compared to the MRE with non-corroded CIPs. Simultaneously, at a higher strain being applied, both MRE samples with non-corroded and corroded CIPs showed reduction trends of the storage modulus, which resulted from the breaking down of the filler network. This reduction trend, alternatively known as the non-linear behavior of strain-dependent dynamic modulus effect, is related to the Payne effect that is attributed to deformation-induced changes in the microstructure of the material. In the meantime, the shorter linear region in the MRE with corroded CIPs are due to the weak particle/matrix interaction, which is due to better interaction of filler/filler (CIPs) through the modification of its particles’ uniform distribution in the matrix.

**Figure 10:**
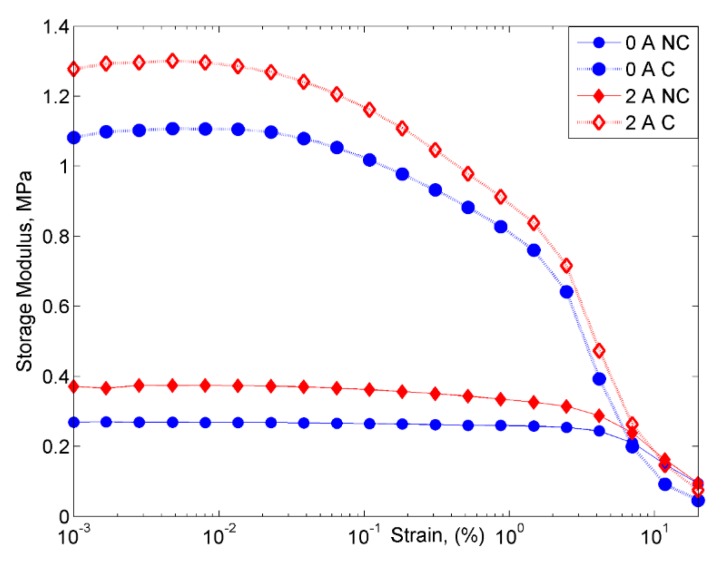
Comparison of storage modulus vs. strain for MRE samples of non-corroded (NC) and corroded (C) CIPs at off- and on-state conditions.

The strain-dependent storage modulus of both MRE samples at off- and on-state conditions are shown in Figure 10.

**Figure 11:**
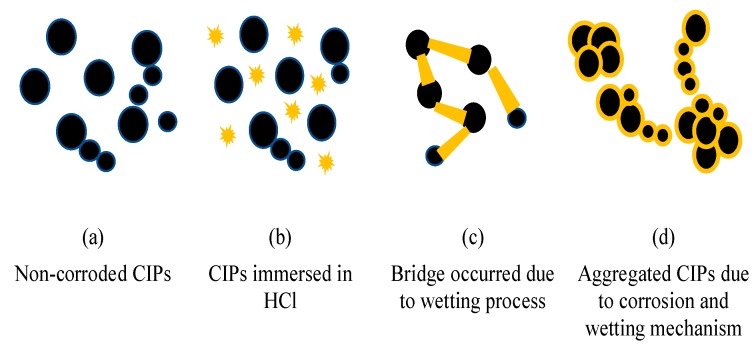
Mechanism of aggregated CIPs due to the corrosion process are illustrated in (**a**) Non-corroded CIPs, (**b**) CIPs immersed in HCl, (**c**) Bridge occurred due to wetting process and (**d**) Aggregated CIPs due to corrosion and wetting mechanism.

The value of initial storage modulus of MRE with non-corroded CIPs without a magnetic field was at 0.26 MPa and the value was higher at an applied current of 2A, which was about 1.08 MPa with a 315% increment. The increased of the initial storage modulus in MRE samples can be explained by a stronger bonding interaction that occurred between the CIPs, which can be illustrated in Figure 11.

As depicted in Figure 11a, the non-corroded CIPs tended to disperse randomly before the mixing process. However, during the immersion process caused by the mixture of HCl, the CIPs experienced other phenomena, which were called the ‘wetting process’ (see Figure 11b). The wetting process is the condition when the HCl has the ability to contact with the solid surface of CIPs, which resulted with a new ‘bridge’ forming between the CIPs as illustrated in Figure 11c, and led to the simultaneous occurrence of the vigorous aggregated CIPs, as shown in Figure 11d. It is also believed that the intermolecular interactions of CIPs will form strong bonding and lead to the higher storage modulus. Therefore, in our study, it is assumed that the particles agglomeration is not destroyed by a low shear mixing process which in this case, meant that the stirrer speed did not appear to affect the agglomeration which occurred in CIPs. These results agree well with the one reported by the Rahmanian et al. [33], as the author used 300 to 510 rpm during the mixing process, and the result revealed that at a higher stirrer speed, the granule size distribution seemed to not be affected. In the meantime, it is also assumed that the agglomeration of CIPs was not affected due to the positive influence by bridging agent (HCl) [34].

**Figure 12:**
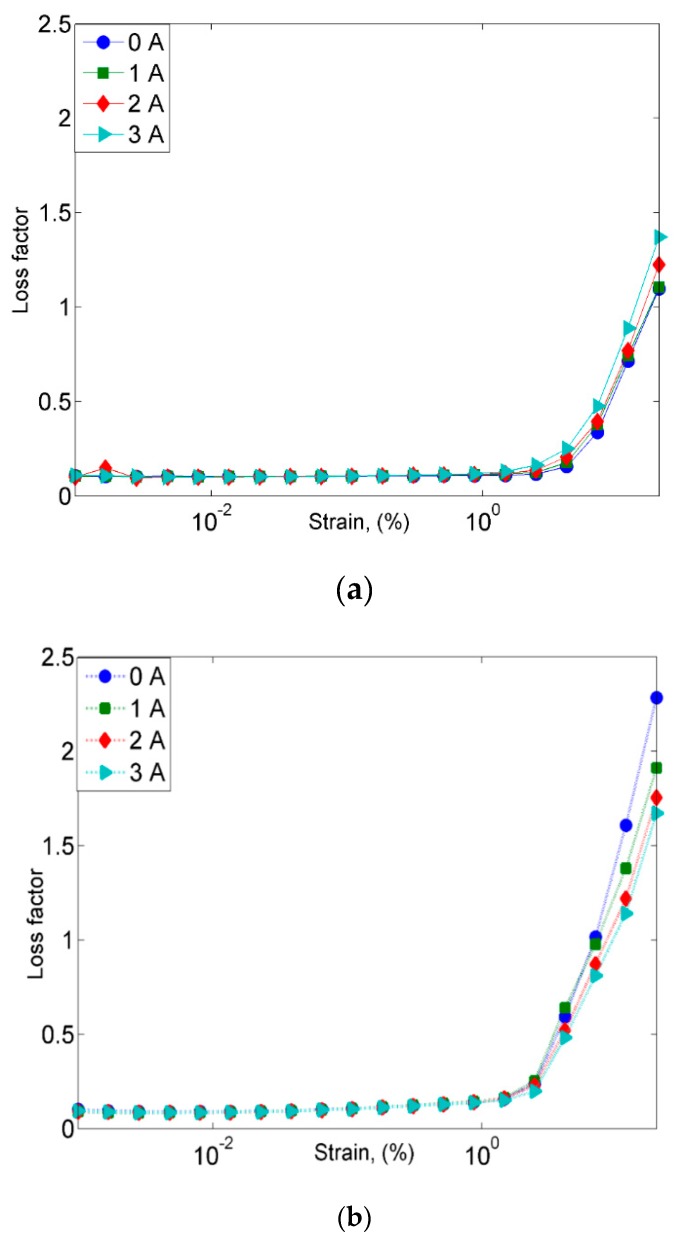
Loss factor vs. strain for (**a**) MRE with non-corroded CIPs and (**b**) MRE with corroded CIPs.

Nonetheless, the macromolecular viscoelasticity or the damping property of MRE is one of the main parameters required in the characterization of material properties. Therefore, it is important to take the loss factor or tan δ (which is defined as the ratio of the loss modulus to the storage modulus) into consideration as a way of gaining information related to the material behavior. The relationship between the loss factor and strain for the MRE with non-corroded and corroded CIPs samples at off- and on-state conditions is shown in Figure 12.

As shown in Figure 12a, the loss factor for the MRE with non-corroded CIPs at a strain of over 2% was observed to have increased up to 1.5 with respect to the strain, while the MRE with corroded CIPs had shown a rapid increase in the loss factor of up to 2.3 at a strain of over 1%. Both MRE samples exhibited almost unchanged loss factors to those at the lower strain. Despite this, the values increased dramatically at the higher strain of 2%. Meanwhile, at a higher strain over 1%, the MRE with non-corroded CIPs exhibited an increment trend of the loss factor, parallel to the increment of the applied magnetic field. Nevertheless, the MRE with corroded CIPs showed a vice versa trend in which with the increment of strain over 2%, the loss factor decreased with the increment of the magnetic field. This trend can be seen in Figure 12b, in which the loss factors of 0, 1, 2 and 3A were 2.3, 1.9, 1.7, and 1.6, respectively. The decreasing trend of the loss factor in MRE with corroded CIPs on the other hand, had directly indicated the significant reduction of energy dissipation in the MRE with corroded CIPs.

#### 2.2.3. Current Sweep Test

**Figure 13:**
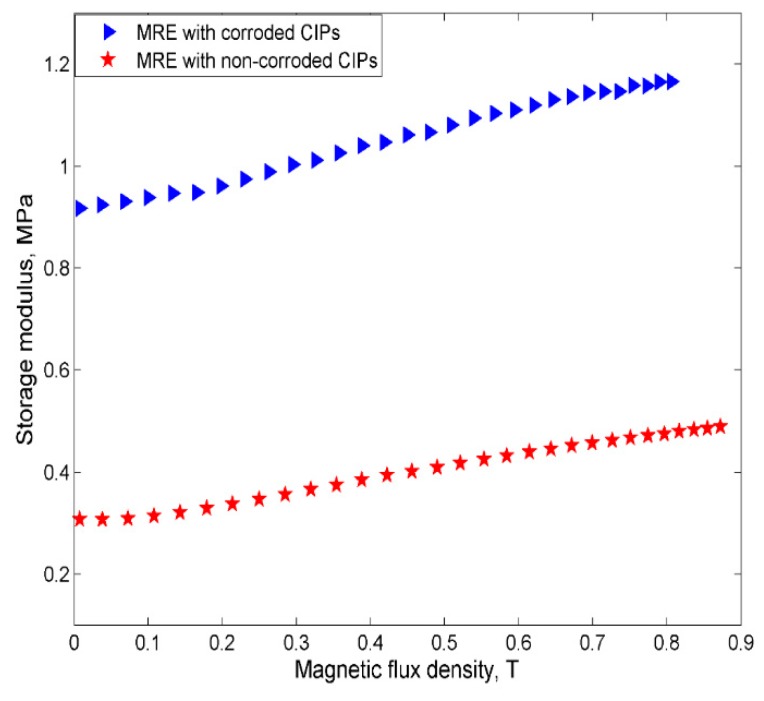
The storage modulus of the MRE samples of non-corroded and corroded CIPs vs. magnetic flux densities.

The relationship between the storage modulus of MRE samples at various magnetic flux densities are measured and presented in Figure 13.

It could be observed that the magneto-induced storage modulus of MRE with non-corroded CIPs had increased from 0.3070 to 0.4887 MPa, while those with corroded CIPs had shown a substantial increase from 0.9168 to 1.1657 MPa. The absolute and MR effect of MRE with both non-corroded and corroded CIPs are illustrated in Table 5.

It is calculated that MR effect of MRE with corroded CIPs was decreased by almost 114% as compared to MRE with non-corroded CIPs. The decreased in MR effect could be due to the higher stiffness level experienced by the MRE with corroded CIPs as the chemical reaction occurred during the immersion of HCl and caused the release of corrosive micro molecules during the mixing process. These micro molecules could have penetrated through the rubber surface and reacted with the molecular chain of the rubber, hence causing a chemical stress relaxation that interferes with the molecular chain and resulting in the stress-softening effects of rubbers. This phenomenon is found to agree well with the other previously conducted studies [14]. Besides the above observable fact, the changes in the outer layer of the CIPs structures as a result of the increased oxygen content in the corroded CIPs had also led to the weakness of the interfacial adhesion strength between the CIPs.

## 3. Materials and Methods

### 3.1. Samples Preparation

#### 3.1.1. Corroded CIPs

**Figure 14:**
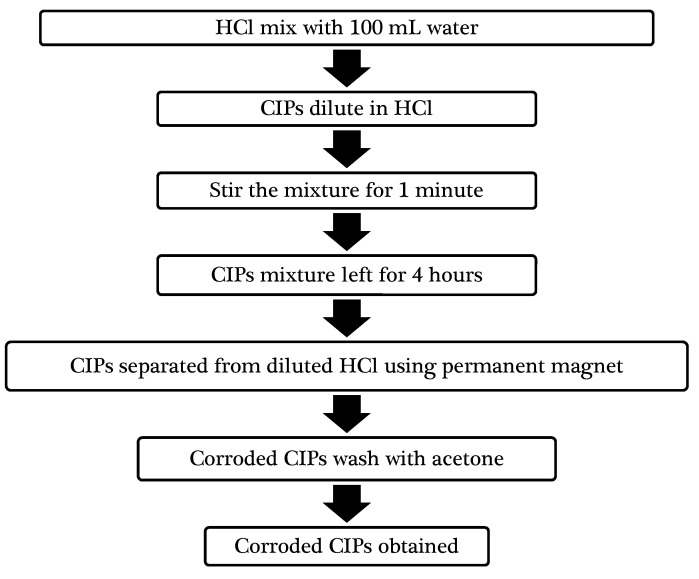
Schematic diagram of the corrosion process of CIPs.

Soft spherical CIPs types (OM) consisting of ≥ 97.8% Fe, produced by BASF; (https://www.basf.com/my/en.html) with an average diameter range of 1-10 µm were used as magnetic particles. While 37% hydrochloric acid (HCl) with analytical reagent (AR) grade (Friendemann Schmidt Chemical; http://www.thermo-line.com/) was utilized for preparing corroded CIPs. The HCl properties and the corrosion process are summarized in Table 6 and Figure 14, respectively.

The amount of hydrochloric acid was 1/19, about 5% relative to water. The dilution process was carried out in a laboratory test chamber. After that, 40 g of CIPs were slowly added into 100 mL of the diluted HCl and the mixture was stirred continuously for 1 min. Some vigorous reaction from the CIPs could be observed during this process. Since a microscopic test conducted by Kolman and Butt (1999) had shown the pitting corrosion of Al composite to have occurred during a 4-h immersion test [35], the CIPs mixture was then left for 4 h. After that, the CIPs were then separated from the diluted HCl by using a permanent magnet and washed several times with acetone to prevent the occurrence of further oxidation from any remaining HCl matrices and water trappings in the CIPs.

#### 3.1.2. MRE Fabrication

Room temperature vulcanization silicon rubber (SR) NS625A with curing agent NS625B (Nippon Steel, Tokyo, Japan were used in the fabrication of two MRE samples through the following three-stage mixing process. 70 wt% of CIPs were mixed with SR for 5 min using mechanical stirrer, and then multi-mix speed dispersed (HSD) with the speed of 250 rpm was performed to achieve a homogenous mixture. Curing agent was subsequently added into the mixture with an SR to curing agent ratio of 95:5 and was stirred continuously for 1 min before being poured into a steel mould (cylinder shape) and cured for 24 h. Using the above process, 20 mm and 1 mm of circular shape and thickness of MRE samples, respectively, were prepared in this work.

#### 3.1.3. MRE Characterization

Morphological observation of MRE samples such as size and shape of before and after corroded CIPs were examined using the field emission scanning electron microscopy (FESEM) equipped with energy dispersive spectroscopy (EDX) (Joel, JSM-7800F, Tokyo, Japan). MRE samples were analyzed via the magnification of 10000× and 16000× at accelerating voltage of 3kV, and the crystallinity of CIPs was examined through X-ray diffractometer (Empyrean, PAN-analytical). A radiation source of Cu Kα radiation of k = 0.154 nm was measured in 2θ between a range of 20° to 100° with a generator setting of 40 mA and 45 kV. The magnetic properties of those CIPs were investigated by using vibrating sample magnetometer (VSM) (Microsense, FCM-10, Lowell, Massachusetts, USA) with a continuous application of magnetic field that has a maximum of 1500 kA/m. Meanwhile, the field-dependent rheological properties of MRE samples were analyzed by using the rheometer (Physica MCR 302, Anton Paar, Graz, Austria) that was equipped with an MR device (MRD 170) in an oscillation state. The test was conducted at a fixed temperature of 25 °C under an oscillating mode, while the magnetic field strength values for each applied current were recorded using a Teslameter. Three sets of data with different oscillation amplitudes and magnetic inputs on the MRE samples were collected from the test. In order to prevent the MRE samples from sliding on the oscillating disk, a pre-load of 15 N was applied during the experimental set-up. For the strain sweep test, the investigation on the storage modulus, the loss modulus, and loss factor were conducted by varying the strain from 0.001 to 20% at five different magnetic fields. In the meantime, the frequency sweep tests were performed from 0.1 to 100 Hz with a constant strain of 0.02%. The applied currents for the strain and frequency sweeps were varied from 0 to 3 A for the strain and frequency sweep with an increment of 1 A, where the magnetic flux densities of 0, 213, 411, and 597 mT. Meanwhile, for the sweep current, the magnetic flux was varied from 0 to 1 T by applying the current from 0 to 5A, respectively.

## 4. Conclusions

In this work, the morphological behaviors and the field-dependent rheological characteristics of MRE with corroded CIPs were experimentally investigated and compared with those of non-corroded CIPs. From the results obtained, the conclusions are listed below:

(1) It has been observed that the increment of almost 23 wt% of oxygen in the corroded CIPs exhibited a higher oxidized condition in the CIPs. From the physiochemical analyses, the corroded CIPs modified the bonding strength between the CIPs as a result of new outer layers formed from the wetting process during the immersion process.
(2) The outer layers of corroded CIPs significantly altered the magnetic behavior of the CIPs by slightly reducing the magnetic saturation of the corroded CIPs by almost 10%.
(3) However, despite the deterioration of the magnetic properties, the rheological properties such as storage modulus of the MRE with corroded CIPs exhibited an increasing trend in both off- and on-state conditions.
(4) The result also revealed the “bridge”, which occurred during the corrosion process, to have enhanced bonding between CIPs by strengthening the connection between the magnetic particles.
(5) On the other hand, the damping properties MRE with non-corroded CIPs displayed a declining trend with the increasing magnetic fields in the frequency sweep, which contradicted with the results obtained from the strain sweep analysis.
(6) Since the MR effect of the corroded CIPs decreased by up to 114% as compared to the non-corroded CIPs, the findings from this study assert that the MRE reactivity can be affected by the chemical reactions of corrosion and the change in the field-dependent rheological properties is quite “unpredictable”.

In the future, deeper research on the motion of the corroded CIPs in MRE and the pH evolution in time and the oxidation rate which can alter the field-dependent rheological properties is required at various environmental conditions to understand the relationship between the chemical reaction and magnetic-response mechanism of the corroded CIPs. In the meantime, the effects of the rubber aging and both the rubber aging and particle corrosion on the MR effect of MRE are other important issues from the practical point of view. This work will be undertaken soon as the second phase of this work.

## Figures and Tables

**Table 1:** Oxygen content of non-corroded and corroded CIPs by EDX.

Sample	Element	Weight %
**Non-corroded CIPs**	Fe	99.79
	O	0.21
**Corroded CIPs**	Fe	77.06
	O	22.94

**Table 2:** Magnetic properties of MRE with non-corroded and corroded CIPs.

Samples	*Ms* (Am^2^/kg)	*Mr* (Am^2^/kg)	*Hc* (kA/m)
MRE with non-corroded CIPs	112.3	1.30	0.70
MRE with corroded CIPs	102.2	1.28	0.68

**Table 3:** Initial and maximum storage modulus of MRE samples with non-corroded and corroded CIPs.

Samples	Initial Storage Modulus				Maximum Storage Modulus			
	Current (A)				Current (A)			
	0	1	2	3	0	1	2	3
MRE with non-corroded particles	0.26	0.29	0.34	0.39	0.38	0.43	0.49	0.55
MRE with corroded CIPs	0.62	0.68	0.74	0.79	1.01	1.05	1.16	1.22

**Table 4:** Initial storage modulus for MRE with non-corroded and corroded CIPs at different current.

Samples	Initial Storage Modulus (MPa)			
	Current (A)			
	0	1	2	3
MRE with non-corroded particles	0.26	0.31	0.37	0.40
MRE with corroded CIPs	1.08	1.17	1.28	1.27

**Table 5:** Initial modulus, absolute and MR effect of MRE samples with corroded and non-corroded CIPs.

Samples	Initial Modulus, G’ (MPa)	Absolute MR Effect, Δ G’	MR Effect (%)
MRE with non-corroded CIPs	0.3070	0.1817	58.19
MRE with corroded CIPs	0.9168	0.2489	27.15

**Table 6:** Basic properties of hydrochloric acid grade AR.

Properties	Values/Limits
**Form**	Liquid
**pH**	<1, at 20 °C
**Relative density**	ca. 1.19 g/cm^3^, at 20 °C
**Water solubility**	Soluble, at 20 °C
**Corrosion**	May corrosive to metals
